# Responding to the workforce crisis: consensus recommendations from the Second Workforce Summit of the American Society of Pediatric Nephrology

**DOI:** 10.1007/s00467-024-06410-9

**Published:** 2024-07-08

**Authors:** Danielle E. Soranno, Sandra Amaral, Isa Ashoor, Meredith A. Atkinson, Gina-Marie Barletta, Michael C. Braun, Joann Carlson, Caitlin Carter, Annabelle Chua, Vikas R. Dharnidharka, Keri Drake, Elif Erkan, Dan Feig, Stuart L. Goldstein, David Hains, Lyndsay A. Harshman, Elizabeth Ingulli, Alexander J. Kula, Mary Leonard, Sudha Mannemuddhu, Shina Menon, Zubin J. Modi, Marva Moxey-Mims, Arwa Nada, Victoria Norwood, Michelle C. Starr, Priya S. Verghese, Darcy Weidemann, Adam Weinstein, Jodi Smith

**Affiliations:** 1 Department of Pediatrics, Indiana University School of Medicine, Indianapolis, IN USA; 2 Department of Bioengineering, Purdue University Weldon School of Engineering, 1044 W. Walnut Street, West Lafayette, IN R4-42146202 USA; 3 Departments of Pediatrics and Biostatistics, Epidemiology and Informatics, Children’s Hospital of Philadelphia and University of Pennsylvania, Perelman School of Medicine, Philadelphia, PA USA; 4 Department of Pediatrics, Harvard Medical School, Boston Children’s Hospital, Boston, MA USA; 5 Department of Pediatrics, Johns Hopkins University School of Medicine, Baltimore, MD USA; 6 Doernbecher Children’s Hospital, Oregon Health & Science University, Portland, OR USA; 7 Department of Pediatrics, Baylor College of Medicine, Houston, TX USA; 8 Department of Pediatrics, Rutgers Robert Wood Johnson Medical School, New Brunswick, NJ USA; 9 Rady Children’s Hospital, University of California San Diego, San Diego, CA USA; 10 Duke Children’s Hospital and Health Center, Durham, NC USA; 11 Washington University in St. Louis, St. Louis, MO USA; 12 University of Texas Southwestern Medical Center, St. Louis, MO USA; 13 Department of Pediatrics, University of Cincinnati College of Medicine, Indianapolis, IN USA; 14 Department of Pediatrics, University of Alabama, Heersink School of Medicine, Birmingham, AL USA; 15 Department of Pediatrics, University of Iowa Stead Family Children’s Hospital, Iowa City, IA USA; 16 Ann & Robert H Lurie Children’s Hospital, Chicago, IL USA; 17 Department of Pediatrics, Stanford University, Stanford, CA USA; 18 East Tennessee Children’s Hospital, Knoxville, TN USA; 19 Department of Pediatrics and Susan B. Meister Child Health Evaluation and Research (CHEAR) Center, University of Michigan, Ann Arbor, MI USA; 20 Department of Pediatrics, Children’s National Hospital/George Washington University SOM, Washington, D.C USA; 21 Department of Pediatrics, Le Bonheur Children’s Hospital, UTHSC, Memphis, TN USA; 22 Department of Pediatrics, University of Virginia, Charlottesville, VA USA; 23 Department of Pediatrics, Division of Nephrology, Children’s Mercy Kansas City, Kansas City, MO USA; 24 University of Missouri–Kansas City School of Medicine, Kansas City, MO USA; 25 Department of Medical Sciences and Pediatrics, Frank H. Netter MD School of Medicine at Quinnipiac University, North Haven, CT USA; 26 Department of Pediatrics, University of Washington, Seattle, WA USA

**Keywords:** Pediatric Nephrology, Workforce crisis, Pediatric sub-specialties, Pay equity, Reimbursement and salary benchmarks, Academic RVUs

## Abstract

**Importance:**

Pediatric patients with complex medical problems benefit from pediatric sub-specialty care; however, a significant proportion of children live greater than 80 mi. away from pediatric sub-specialty care.

**Objective:**

To identify current knowledge gaps and outline concrete next steps to make progress on issues that have persistently challenged the pediatric nephrology workforce.

**Evidence review:**

Workforce Summit 2.0 employed the round table format and methodology for consensus building using adapted Delphi principles. Content domains were identified via input from the ASPN Workforce Committee, the ASPN’s 2023 Strategic Plan survey, the ASPN’s Pediatric Nephrology Division Directors survey, and ongoing feedback from ASPN members. Working groups met prior to the Summit to conduct an organized literature review and establish key questions to be addressed. The Summit was held in-person in November 2023. During the Summit, work groups presented their preliminary findings, and the at-large group developed the key action statements and future directions.

**Findings:**

A holistic appraisal of the effort required to cover inpatient and outpatient sub-specialty care will help define faculty effort and time distribution. Most pediatric nephrologists practice in academic settings, so work beyond clinical care including education, research, advocacy, and administrative/service tasks may form a substantial amount of a faculty member’s time and effort. An academic relative value unit (RVU) may assist in creating a more inclusive assessment of their contributions to their academic practice. Pediatric sub-specialties, such as nephrology, contribute to the clinical mission and care of their institutions beyond their direct billable RVUs. Advocacy throughout the field of pediatrics is necessary in order for reimbursement of pediatric sub-specialist care to accurately reflect the time and effort required to address complex care needs. Flexible, individualized training pathways may improve recruitment into sub-specialty fields such as nephrology.

**Conclusions and relevance:**

The workforce crisis facing the pediatric nephrology field is echoed throughout many pediatric sub-specialties. Efforts to improve recruitment, retention, and reimbursement are necessary to improve the care delivered to pediatric patients.

**Graphical Abstract:**

**Figure:**
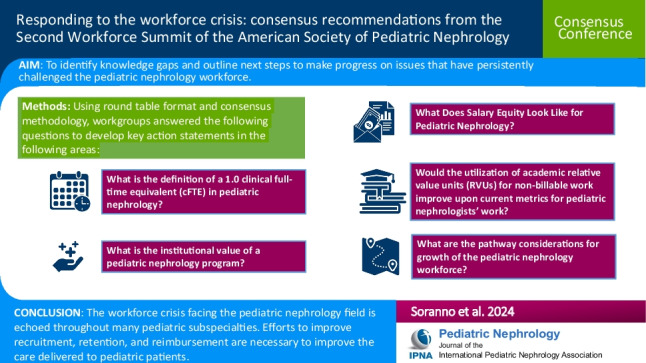
A higher resolution version of the Graphical abstract is available as Supplementary information.

**Supplementary Information:**

The online version contains supplementary material available at 10.1007/s00467-024-06410-9.

## Introduction

The American Society of Pediatric Nephrology (ASPN) is the leading voice of pediatric nephrology in North America. Its primary goal is to advance the care for children, adolescents, and young adults with kidney disease through advocacy, education, research, and workforce development. Compelled by a persistent and growing pediatric nephrology workforce crisis, the ASPN convened a second Workforce Summit (Workforce Summit 2.0). The first Workforce Summit, held in 2019, demonstrated the urgent need for equitable reimbursement as well as recruitment and retention strategies to ensure a sustainable, robust, and diverse pediatric nephrology workforce [1]. As a result, the ASPN has made concerted policy and advocacy efforts; however, the workforce crisis not only persists but has worsened in the past 4 years [1]. In the USA, pediatric nephrology fellowships are 3 years in duration, with approximately 1/3 of the time focused on clinical care and the remaining time focused on scholarly projects (e.g., basic science research, clinical research, and quality improvement projects). Upon completion of training, most nephrologists enter the academic workforce, and despite the emphasis on research and scholarly projects during their training, the majority of nephrologists spend the bulk of their time performing clinical care. Despite the length of training, which is equivalent to many other pediatric sub-specialty fellowships (e.g., intensive care, neonatology, and cardiology), the salary benchmarks of pediatric nephrologists, controlling for academic rank and geographic region, are lower than most other pediatric sub-specialties. Nephrologists, however, are not alone in this as several pediatric sub-specialties (e.g., infectious disease) earn lower salaries than general pediatricians [2].

Within this landscape, in 2023 46% of pediatric nephrology fellowship positions went unfilled, and pediatric nephrology positions remain the lowest filled of all pediatric sub-specialties from 2014 to 2022 at only 65.7% final fill rate [3, 4]. A detailed projection of future workforce needs by the American Board of Pediatrics anticipates growing demand and widening geographic disparities in the pediatric nephrology workforce from 2020 to 2040 [5]. The objectives of the Summit were to identify current knowledge gaps and outline concrete next steps to make progress on issues that have persistently challenged the pediatric nephrology workforce.

The committee recognizes that other pediatric sub-specialties face similar challenges in workforce recruitment, retention, and reimbursement [2, 6, 7]. Children receive optimal care when they have access to providers who have been trained specifically to care for children, yet an estimated 2–53% of children live > 80 mi. away from pediatric sub-specialty care [8–12]. Advocacy beyond one sub-speciality is not only warranted but essential in order to optimize the care that pediatric community provides to children [13]. The committee also recognizes that some of the workforce issues discussed herein are specific to the unique practice environment of the USA (e.g., reimbursement/salary); however, many of the issues are more broadly applicable to pediatric nephrologists practicing around the world (e.g., garnering institutional support and recruitment of trainees) [14].

## Methods

The Workforce Summit 2.0 employed a round table format and methodology for consensus building using adapted Delphi principles [15, 16]. Content domains were identified via input from the ASPN’s Workforce Committee, 2023 Strategic Plan survey, Pediatric Nephrology Division Directors survey, and ongoing feedback from members. The organizing committee comprised the ASPN President and Workforce Committee Chair. In order to create the content domains and organize the working groups, the organizing committee collated the feedback and identified themes. Five themes were identified, including definition of full-time effort, non-billable work, obtaining institutional support for a robust pediatric nephrology service, salary equity, and recruitment and retention of the workforce. The key controversy was identified for each domain and turned into a question for the working group to answer. The organizers invited 28 faculty comprising diverse career types according to their topic-related expertise. Diverse interpersonal representation was also sought out, including considerations given to gender, age, race, ethnicity, and LGBTQ + status. Once assembled, faculty was separated into the five working groups to focus on the assigned question for their domain: (1) What is the definition of a 1.0 clinical full-time equivalent (cFTE) in pediatric nephrology?, (2) Would the utilization of academic relative value units (RVUs) for non-billable work improve upon current metrics for pediatric nephrologists’ work?, (3) What is the institutional value of a pediatric nephrology program?, (4) What does salary equity look like for pediatric nephrology?, and (5) What are the pathway considerations for growth of the pediatric nephrology workforce? Working groups met prior to the Summit via conference calls to conduct an organized literature review and establish key questions to be addressed. The Summit was held in-person in Philadelphia, Pennsylvania in November 2023. During the Summit, work groups presented their preliminary findings, and the at-large group developed the key action statements and future directions presented herein.

## Results

### Group 1: What is the definition of a 1.0 cFTE in pediatric nephrology?

#### Consensus statement 1a

Clinical full time equivalent (cFTE) includes all billable and non-billable activities related to providing high-quality clinical care for children with kidney disease.

#### Consensus statement 1b

Each pediatric nephrology program determines the appropriate makeup of inpatient and outpatient work that best suits their specific patient population and clinical mission and balances the priorities of providing safe and effective care with workforce equity and well-being.

##### Rationale

The variability of the clinical work of pediatric nephrologists in different hospital systems renders it difficult to quantify and standardize cFTE using typical calculations (i.e., shifts and clinics) [17]. The work performed by a pediatric nephrologist may include procedural and cognitive components, inpatient coverage and outpatient clinics, and overnight call with potential for life-saving emergency procedures. In addition, the high medical complexity of pediatric nephrology patients requires multidisciplinary collaboration, attention to primary and preventive care designed to slow the progression of kidney disease, and frequent detailed patient and family conversations to ensure sufficient understanding of their child’s disease. The 24-h call coverage entails significant after-hours physician input, often with provision of emergent dialysis which requires physician presence during treatment and decision-making about organ suitability for pediatric transplant candidates. The relative lack of compensation proportional to the perceived workload has been identified as an important root cause of the pediatric nephrology workforce crisis [1, 18, 19]. We recommend that a comprehensive analysis be performed to describe the time and effort required for a discrete block of clinical work that encompasses both inpatient and outpatient responsibilities. This analysis would holistically evaluate the work required for a standard 4-h half-day outpatient pediatric nephrology clinic. Specific measures for outpatient analysis would consist of time spent during direct, face-to-face patient interaction, and the additional workload outside the exam room relevant to patient care, including clinic preparation time, order entry, post-clinic documentation, and laboratory/imaging management. A similar analysis can be performed for inpatient pediatric nephrology service coverage. Specific measures for inpatient analysis would include the time spent during direct, face-to-face patient interaction, documentation with laboratory/imaging management, hand-off communication, and after-hours call burden including frequency of transplant organ offer calls for patients awaiting kidney transplantation, and emergent dialysis which requires physician presence during treatment. Data obtained from these analyses could be compared to adult nephrology to better understand the relative workload. A pilot study is also proposed using electronic health record (EHR) data analytics and self-report, as well as time-motion analysis, to collect data in granular detail. Additionally, we recommend collecting work data related to key clinical leadership and/or administrative roles (Dialysis Medical Director, Transplant Medical Director, Acute Care Nephrology Director, etc.) which should be included in the FTE description. The committee recognizes that the practice of pediatric nephrology varies broadly across institutions and regions. Important programmatic variables that can impact workload include the number of practicing nephrologists at the program, presence of fellows, residents, and/or advanced practice providers, catchment area and population size, hospital volumes and case mix index, presence/availability of pediatric dialysis and kidney transplantation, local resources, and presence of multidisciplinary programs that require pediatric nephrology expertise (i.e., level 1 Trauma designation, solid organ and bone marrow transplant programs, high-risk obstetric delivery services and level 1 NICU, kidney transplant volume, and the size of the outpatient peritoneal and hemodialysis populations). Given the broad variability that can exist between programs, we caution against the use of benchmarking metrics to define clinical work [20]. In addition, an attempt to determine a universal 50th percentile RVU:cFTE benchmark may perpetuate a “race to the bottom” in which clinicians would be incentivized to spend less time per patient than their peers and may ultimately degrade the quality of care provided [20]. Instead, we propose that individual pediatric nephrology programs use available data to perform detailed and transparent internal work analysis specific to their program and clinical needs. A “one-minus” model could be utilized, in which basic principles describe time allocation to clinical, research, teaching, and administrative activities which sum up to 1.0 FTE [21]. This methodology could subsequently be used to create division-specific worksheets to determine cFTE components which are equitable, fair, and transparent. Example templates could be created by professional societies as “starting points” for small, medium, and large-sized programs through the use of the detailed time-work analyses while recognizing that adaptation of worksheets to fit the needs of the local environment is key to creating a sustainable model for all parties involved.

### Group 2: Would the utilization of academic relative value units (RVUs) for non-billable work improve upon current metrics for pediatric nephrologists’ work?

#### Consensus statement 2a

The effort pediatric nephrologists spend on academic non-revenue generating pursuits including educational, research, and administrative activities can be quantified and factored into determining their available capacity for providing clinical care.

#### Consensus statement 2b

A standardized rubric to track achievements in non-clinical academic activities in the areas of education, research, quality improvement, administrative leadership, and division citizenship provides a fair and consistent approach to incentive compensation, if applicable.

##### Rationale

Academic physicians routinely dedicate effort to non-revenue generating activities beyond direct patient care. For pediatric nephrologists who practice in academic medical centers, this is an implied expectation to advance core institutional missions including clinical, research, educational, and advocacy goals [22]. While patient care clinical activities are quantified by well-established Current Procedural Terminology (CPT®) codes [23], compensation for academic pursuits varies [24]. Certain aspects of the pediatric nephrologist’s academic endeavors may be linked to financial compensation and/or protected time. Those include governmental or foundational grant-supported research activities, Accreditation Council for Graduate Medical Education (ACGME)-accredited fellowship program director role [25] and dialysis medical director roles [26]. However, most academic activities are completed at the physician’s own discretion including clinical research activities, mentoring, resident and medical student education, participation in quality improvement projects, and other division, hospital level, or organizational committee leadership roles. Failure to recognize the effort physicians invest into these non-clinical efforts risks physician burnout, job dissatisfaction, and subsequent attrition from the field [27]. While physician compensation models vary and are institution-specific, many may include an end of year incentive payment model that rewards physician clinical productivity [28, 29]. Failure to adapt incentive payment models to quantify and reward academic efforts and move away from purely clinical RVU-based metrics risks stifling academic innovation by shifting physician behavior to focus on clinical revenue-generating patient activities over academic endeavors [30].

### Group 3: What is the institutional value of a pediatric nephrology program?

#### Consensus statement 3a

Pediatric nephrologists contribute to institutional financial margins in ways that are separate from work RVUs (wRVUs), thus the wRVU system undervalues the effort and indirect income generated by pediatric nephrologists.

#### Consensus statement 3b

Availability of a pediatric nephrologist is a prerequisite to many of the high-value medical services offered by institutions.

#### Consensus statement 3c

As we move towards quality- and value-based care models, pediatric nephrologists will play a critical role in the financial well-being of medical institutions.

##### Rationale

Current wRVU metrics used to estimate the financial value of pediatric nephrologists to their institutions are flawed. The work of pediatric nephrologists, like many less procedurally oriented specialties, is undervalued by the wRVU system [18, 31]. While caring for their primary and consult patients, pediatric nephrologists generate orders and referrals for laboratory testing, medical imaging, surgical procedures, and sub-specialty consultation — none of which are captured by wRVU [18]. Pediatric nephrologists enable institutions to offer a diverse array of medical services. This is especially true for high-margin service lines such as neonatal intensive care, cardiac surgery, solid organ transplant, and oncology/bone marrow transplantation. Furthermore, the care of critically ill children has been incentivized for institutions due to these high-margin service lines. While pediatric nephrologists perform critical roles in the care of these patients (e.g., dialysis procedures or care after organ transplantation), the downstream revenue supports the primary services (critical care) much more than consulting services. Regulatory bodies, accreditation entities, society guidelines, and quality metrics such as the US News World Report rankings track the availability of pediatric nephrology services and kidney replacement therapy programs to determine designations for clinical services at the highest level of care. As more payers move to value-based reimbursement, the increase in cost-savings and efficiency provided by pediatric nephrologists should be recognized [32, 33]. Furthermore, institutions invested in pediatric care would benefit from a heightened awareness of the financial repercussions that may result if the shortage of pediatric nephrologists continues to worsen [1]. In the outpatient setting, the shortage of pediatric nephrologists has resulted in long travel distances for many patients to obtain pediatric nephrology care. This has led to an increase in outreach clinics to better serve the community; however, such clinics place a burden on the workforce in the form of significant travel time, time away from family, and working in clinic environments that may not be able to provide the same level of service as the main practice clinic (e.g., urine microscopy).

### Group 4: What does salary equity look like for pediatric nephrology?

#### Consensus statement 4a

Compensation for pediatric nephrologists will represent the value of kidney care to their organization, including complexity of caring for patients across the spectrum of pediatric care. Further, compensation will reflect the value added by pediatric nephrologists in support of hospital and programmatic missions including margin positive services that require pediatric nephrology expertise.

#### Consensus statement 4b

Reimbursement for provision of sub-specialty care to children with kidney disease will accurately reflect the time and effort required to address their complex, multi-system disease manifestations, such as growth, development, and nutritional needs. In such a system, RVU would be adjusted to reflect the complexity and demands of care. This alignment is critical to prevent physician burnout and sustain the workforce.

##### Rationale

In the current fee-for-service system, care for children with kidney disease is neither sufficiently valued nor appropriately compensated [1]. The lower compensation for high workload contributes to decreasing trainee interest in pediatric nephrology [34, 35] and affects recruitment and retention of under-represented minorities and non-financially advantaged individuals. Compensation for pediatric nephrologists should be both representative of the value of pediatric kidney care to their organization and reflective of their sub-speciality training. The recent National Academies of Science, Engineering, and Medicine (NASEM) Committee Report on the Pediatric Subspecialty Workforce and Its Impact on Child Health and Well-Being focused in part on the accurate reflection of the time and effort required to care for pediatric sub-specialty children [3]. Future iterations of payment systems and reimbursement should reduce financial disincentives to sub-specialty training and consider the unique value added of pediatric nephrologists both to individual patient care and health systems. For example, high revenue generating programs such as critical care, stem cell transplantation, and cardiothoracic surgery all rely on the expertise of pediatric nephrologists and dialytic therapies [36, 37]. Proposed solutions include increased pediatric representation on agencies that determine current procedural terminology coding and reimbursement. Payment structure should move away from targeting set national benchmarking metrics (often creating a self-perpetuating cycle) and instead focus on value added. This will require the deliberate action by pediatric department chairs, children’s hospitals/health system chief executive officers, and medical college deans to meet the needed investment in increased compensation benchmarks for pediatric sub-specialties [2, 5]. Children with kidney disease present added complexity given their age and the importance of growth and development along with other complex care needs. In the USA, the Centers for Medicare & Medicaid Services has recently recognized this complexity by providing enhanced reimbursement for pediatric chronic dialysis care through the use of a 30% add-on payment per treatment for pediatric dialysis patients [38]. Broadening this approach for other high-intensity pediatric kidney disease services (such as advanced pre-dialysis chronic kidney disease and acute dialysis in the inpatient setting) should be considered.

### Group 5: What are the pathway considerations for growth of the pediatric nephrology workforce?

#### Consensus statement 5a

Stronger engagement of pediatric nephrologists with trainees throughout undergraduate medical education and during early pediatric residency may increase interest in a career in pediatric nephrology.

#### Consensus statement 5b

Flexibility in fellowship length and design with individualized pathways will encourage more residents to pursue pediatric nephrology, improve training experience, and potentially reduce the debt burden associated with the mandatory 3-year training.

#### Consensus statement 5c

Retention in the existing workforce may be improved by efforts of the ASPN towards incentivizing clinical and research work, improving work-life integration, and increasing remuneration.

##### Rationale

Multiple factors influence a medical student’s decision to choose pediatrics and a pediatric resident to choose pediatric nephrology, including exposure to the subject early on, perceived difficulty of the subject, having role models and mentors in pediatric nephrology, and consideration of lifestyle and earning potential [22, 39–41]. Pediatric nephrology divisions will benefit from dedicated faculty in the division who can intentionally work with trainees across all levels to improve exposure to the subject and provide positive role models for careers in the field. Pediatric sub-specialization is financially disincentivized for trainees, as pediatric sub-specialization both delays completion of training and decreases lifetime earning potential [42]. Notably, this is not the case with adult sub-specialization [2, 43]. Additionally, the length of fellowship training and the rigid template requiring mandatory research and scholarly activity may be a deterrent for some trainees. The three-year training pathway also increases the debt burden of education and training, which combined with the relatively lower salaries leads to significant loss of earning potential [40, 44]. In a survey of almost 800 physicians in their second or third year of pediatric sub-specialty fellowship in the USA in 2007, 52% (*n* = 390) would have chosen a 2-year fellowship with less research or scholarly activity [45]. More recently, in another survey by the American Association of Pediatrics, almost 1500 fellows responded in favor of reducing the training duration to less than 3 years, or having a shorter duration track for those who planned to pursue a clinical path, and a longer one for those pursuing research [46]. The NASEM Report recommended that the ACGME and American Board of Pediatrics develop and evaluate alternative fellowship training requirements and pathways, including a 2-year option for those who wish to pursue a clinically-focused career. ASPN, with its in-depth understanding of the challenges facing the pediatric nephrology workforce, needs to be a part of this restructuring [3]. Longitudinal data to understand the impact of such a change on the composition of the workforce merits collection. Finally, a concerted effort at multiple levels is needed to understand the reasons for attrition from the pediatric nephrology workforce and to implement strategies to improve retention [22, 47]. This includes incentivizing fellows to complete pediatric nephrology training utilizing loan repayment plans and visa sponsorships for international medical graduates. This also includes other interventions to optimize work-life integration, like flexible work schedules, utilization of telehealth for urgent after-hours dialysis initiation, increased engagement of advanced practice providers, and working effectively with general pediatric practitioners to improve referral guidelines to pediatric nephrology, and thus share the workload [48]. Efforts to streamline maintenance of certification may also reduce attrition for nephrologists who may otherwise consider staying in the workforce longer. Finally, the pediatric neprhrology community may benefit from collecting data from nephrologists who decide to leave the workforce earlier than anticipated.

## Limitations

While the faculty who participated in the Summit represent a diverse group of pediatric nephrologists, we note that a key limitation was that some of the issues addressed are specific to providers practicing in the USA, with associated unique reimbursement issues. However, most topic areas have broader implications and many of these topics also apply to other pediatric sub-specialties [49, 50]. We therefore anticipate that our consensus recommendations may prove useful to the larger international pediatric community.

## Discussion

The Workforce Summit 2.0 consensus statements summarize the key issues facing the pediatric nephrology workforce and serve to guide next steps the community may take to strengthen the ability of our community to care for children with kidney disease. Table 1 summarizes the consensus statements from each working group and associated action items. The working group focused on defining a 1.0 cFTE proposes to undertake an observational study in which pediatric nephrologists report the billable and non-billable work that goes into a half-day outpatient nephrology clinic and a week of inpatient nephrology service. The group focused on academic RVU’s proposes to create a rubric that will credit providers with the non-clinical work they perform in support of the academic mission. The working group focused on describing the institutional value of a pediatric nephrology program has proposed a White paper summarizing the critical role that a robust nephrology program provides to other, more incentivized, service lines. This group also intends to demonstrate the potential cost to institutions if they were unable to maintain a pediatric nephrology service. The working group focused on salary equity proposes increased transparency on salary within the ASPN community and intends to post salary metrics on the members’ webpage. And building on the recent reimbursement victory for children with kidney failure, the group will work with other sub-specialties to demonstrate that children who require sub-specialty care are more complex than their adult counterparts, and warrant increased reimbursement based upon the higher level of complexity. And lastly, the working group focused on recruitment and retention of the workforce plans to establish an exit interview system for pediatric nephrologists who leave the workforce early and will continue to advocate for flexibility in the duration of fellowship, depending on the career goals of the trainee. This group will also work to clarify standard operating procedures and referral plans between the primary care providers on the front lines and the pediatric nephrology community in order to streamline referrals.

**Table 1:** Summary of consensus statements and action items

Working group	Consensus statements	Action items
1. What is the definition of a 1.0 clinical full-time equivalent (cFTE) in pediatric nephrology?	a) Clinical FTE (cFTE) includes all billable (patient-facing) and non-billable activities related to providing high-quality clinical care for children with kidney disease b) Each pediatric nephrology program determines the appropriate makeup of inpatient and outpatient work that best suits their unique patient population and clinical mission	• Working group to perform survey and outline consensus definition of the cFTE equivalent of one half-day outpatient nephrology clinic and week of inpatient pediatric nephrology service
2. Would the utilization of academic relative value units (RVUs) for non-billable work improve upon current metrics for pediatric nephrologists’ work?	a) The effort pediatric nephrologists spend on academic non-revenue generating pursuits including educational, research, and administrative activities can be quantified and factored into determining their available capacity for providing clinical care b) A standardized rubric to track achievements in non-clinical academic activities in the areas of education, research, quality improvement, administrative leadership, and division citizenship provides a fair and consistent approach to incentive (bonus) compensation, if applicable	• Working group, in partnership with representatives from each of the ASPN committees, to establish a comprehensive rubric for determining bonus/incentive payments
3. What is the institutional value of a pediatric nephrology program?	a) Pediatric nephrologists contribute to institutional financial margins in ways that are separate from work RVUs (wRVUs), thus the wRVU system undervalues the effort and indirect income generated by pediatric nephrologists b) Availability of a pediatric nephrologist is a prerequisite to many of the high-value medical services offered by institutions c) As we move towards quality and value-based care models, pediatric nephrologists will play a critical role in the financial well-being of medical institutions	• Working group to draft and submit a White paper compiling the various service lines dependent upon a functioning pediatric nephrology program • Working group to investigate the opportunity cost to institutions without a robust pediatric nephrology program
4. What does salary equity look like for pediatric nephrology?	a) Compensation for Pediatric Nephrologists will represent the value of pediatric kidney care to their organization, including complexity of caring for patients across the spectrum of pediatric care. Further, compensation will reflect the value added by pediatric nephrologists in support of hospital and programmatic missions including margin positive services that require pediatric nephrology expertise b) Reimbursement for provision of sub-specialty care to children with kidney disease will accurately reflect the time and effort required to address their complex, multi-system disease manifestations, such as growth and development and nutritional needs. In such a system, RVU would be adjusted to reflect the complexity and demands of care. This alignment is critical to prevent physician burnout and sustain the workforce	• The ASPN will post salary metrics on the members website • The working group will work to establish the proof of principle that pediatric sub-specialty patients are more complex than their adult counterparts. This work will be used to advocate for a reassessment in reimbursement
5. What are the pathway considerations for growth of the pediatric nephrology workforce?	a) Stronger engagement of pediatric nephrologists with trainees throughout undergraduate medical education and during early pediatric residency may increase interest in a career in pediatric nephrology b) Flexibility in fellowship length and design with individualized pathways will encourage more residents to pursue pediatric nephrology, improve training experience, and potentially reduce the debt burden associated with the mandatory 3-year training c) Retention in the existing workforce may be improved by efforts of the American Society of Pediatric Nephrology towards incentivizing clinical and research work, improving work life integration, and increasing remuneration	• The ASPN will establish an exit interview opportunity for faculty leaving the workforce early • The ASPN will advocate for increased flexibility in fellowship training duration • The working group will utilize the NASEM report to establish comprehensive referral guidelines for generalists seeking pediatric nephrology consultation

## Conclusion

The purpose of this Summit was to create concrete steps for improvement in areas crucial to workforce recruitment, retention, and resiliency. These consensus statements outline key areas of focus to improve the sustainability of the pediatric nephrology workforce and many align with issues facing other pediatric sub-specialties. Concerted efforts along these lines may therefore help address workforce challenges not just within pediatric nephrology but also among other pediatric sub-specialties. Improving the strength and resiliency of the pediatric nephrology workforce — and pediatric sub-specialties in general — improves the value and care provided to children.

## Supplementary Information

Below is the link to the electronic supplementary material.

Graphical Abstract (PPTX 553 KB)
