# Supplementary material for: Responding to the workforce crisis: consensus recommendations from the Second Workforce Summit of the American Society of Pediatric Nephrology
Source: Pediatr Nephrol. 2024 Jul 8;39(12):3609–19. doi: 10.1007/s00467-024-06410-9 (PMC11511730; doi:10.1007/s00467-024-06410-9)
Supplement: Supplementary file 1 — (PPTX 553 KB) [file 467_2024_6410_MOESM1_ESM.pptx]

## Slide 1
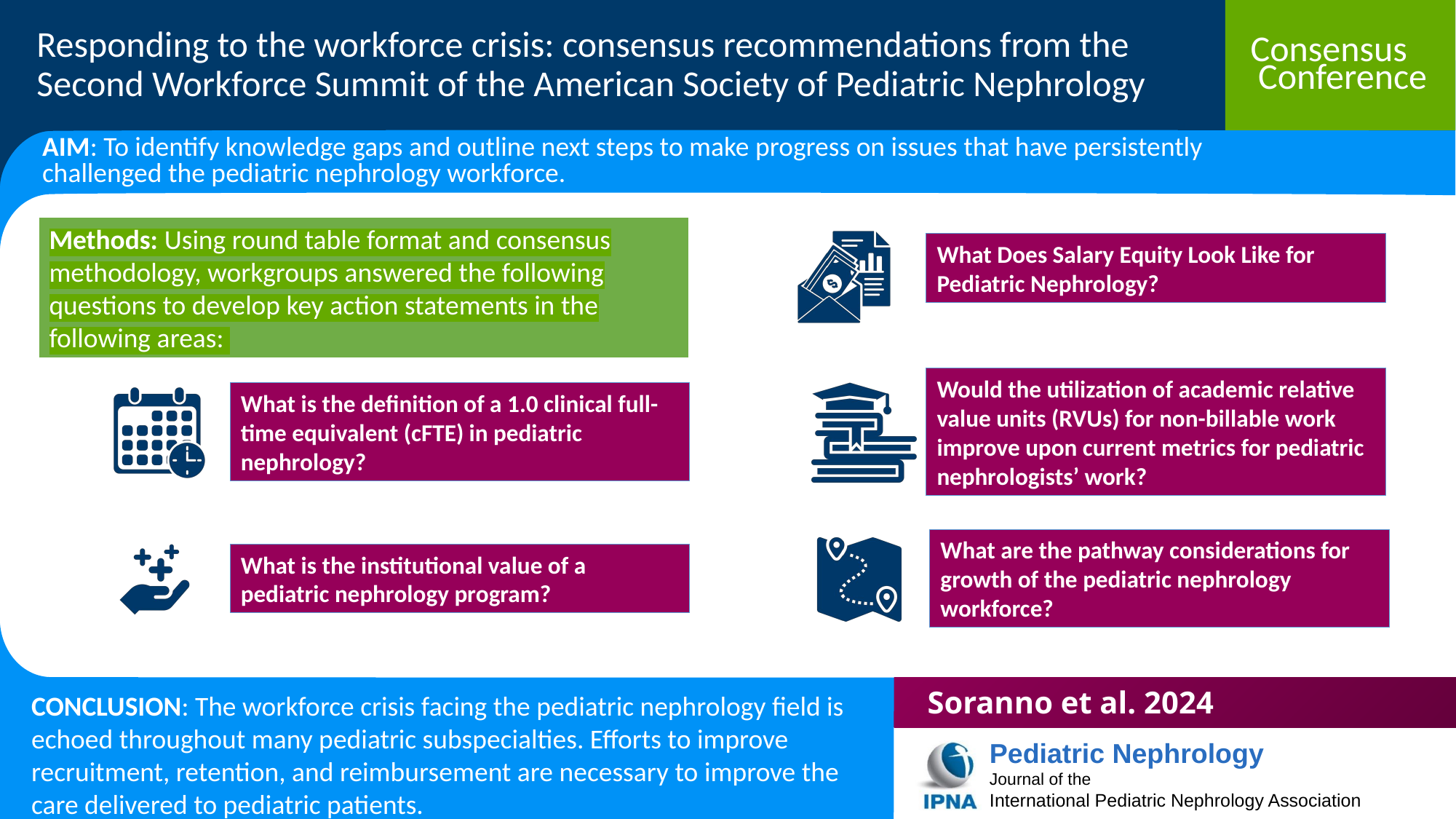

Responding to the workforce crisis: consensus recommendations from the Second Workforce Summit of the American Society of Pediatric Nephrology
AIM: To identify knowledge gaps and outline next steps to make progress on issues that have persistently challenged the pediatric nephrology workforce.
Methods: Using round table format and consensus methodology, workgroups answered the following questions to develop key action statements in the following areas:
What Does Salary Equity Look Like for Pediatric Nephrology?
Would the utilization of academic relative value units (RVUs) for non-billable work improve upon current metrics for pediatric nephrologists’ work?
What is the definition of a 1.0 clinical full-time equivalent (cFTE) in pediatric nephrology?
What are the pathway considerations for growth of the pediatric nephrology workforce?
What is the institutional value of a pediatric nephrology program?
Soranno et al. 2024
CONCLUSION: The workforce crisis facing the pediatric nephrology field is echoed throughout many pediatric subspecialties. Efforts to improve recruitment, retention, and reimbursement are necessary to improve the care delivered to pediatric patients.
